# Pulmonary Tumor Thrombotic Microangiopathy as a Cause of Pulmonary Hypertension

**DOI:** 10.1016/j.jaccas.2021.04.013

**Published:** 2021-07-07

**Authors:** Benjamin P. Schwartz, Philip Tracy, Stephanie Hon, Harrison W. Farber, James E. Udelson

**Affiliations:** a Department of Medicine, Tufts Medical Center, Boston, Massachusetts, USA; b Division of Pulmonary, Critical Care and Sleep Medicine, Tufts Medical Center, Boston, Massachusetts, USA; c Division of Cardiology and the CardioVascular Center, Tufts Medical Center, Boston, Massachusetts, USA

**Keywords:** chronic thromboembolic pulmonary hypertension, CTEPH, PTTM, pulmonary hypertension, pulmonary tumor thrombotic microangiopathy, PTTM, pulmonary tumor thrombotic microangiopathy

## Abstract

Pulmonary tumor thrombotic microangiopathy is a rare entity, often diagnosed postmortem. We describe a patient with signs and symptoms of pulmonary hypertension secondary to metastatic cholangiocarcinoma with invasion of the myocardium and pulmonary vasculature, and highlight the diagnostic challenges and therapeutic limitations of this disease. (**Level of Difficulty: Intermediate.**)

## Central Illustration

**Figure:**
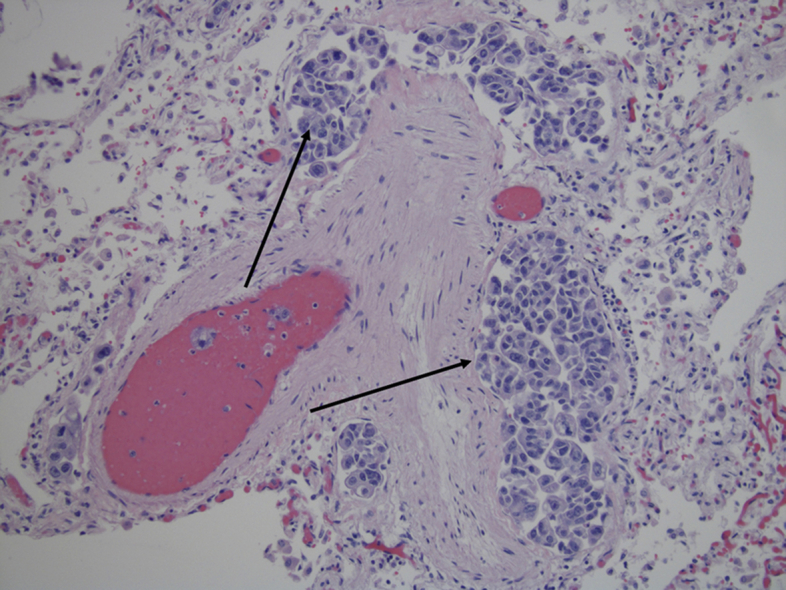

A 64-year-old man presented with 6 months’ history of progressive fatigue, dyspnea, and exercise intolerance. The patient had a past medical history of obesity, obstructive sleep apnea, hypertension, hyperlipidemia, and type 2 diabetes.

On examination, oxygen saturation was 91% while using 2 l of oxygen by nasal cannula. On cardiac exam, there was a pronounced P2 component without right ventricular heave. Pulmonary exam was normal. Jugular venous pressure was elevated to 10 cm of water, and trace bilateral lower extremity edema was present.

Transthoracic echocardiogram (Video 1) was suggestive of pulmonary hypertension, with a severely dilated right ventricle and estimated right ventricular systolic pressure of >70 mm Hg. On laboratory evaluation, there was a normal troponin-T, elevated N-terminal pro–B-type natriuretic peptide of 1,682 pg/ml, modest liver chemistry elevations, and a D-dimer of 2,503 ng/ml. A broad panel of rheumatologic autoantibodies and a SARS-CoV-19 test were negative. Computed tomography pulmonary angiography demonstrated mediastinal and retroperitoneal lymphadenopathy without pulmonary embolism. A ventilation/perfusion scan was read as intermediate-to-high probability for chronic thromboembolic disease.

Right heart catheterization was consistent with severe pre-capillary pulmonary hypertension (right atrial pressure 5 mm Hg, pulmonary artery systolic pressure 71 mm Hg, pulmonary artery diastolic pressure 24 mm Hg, mean pulmonary artery pressure 42 mm Hg, pulmonary capillary wedge pressure 7 mm Hg, cardiac index 1.8 l/min/m^2^ by thermodilution, pulmonary vascular resistance 653 dyne · s · cm^5^). Left heart catheterization demonstrated a 30% stenosis in the right coronary artery and a 60% stenosis in the mid-left anterior descending coronary artery. Pulmonary arteriogram was planned, but the patient had a cardiac arrest before it could be performed; resuscitative measures were unsuccessful. Telemetry tracings demonstrated junctional escape rhythm that devolved into pulseless electrical activity.

**Figure 1:**
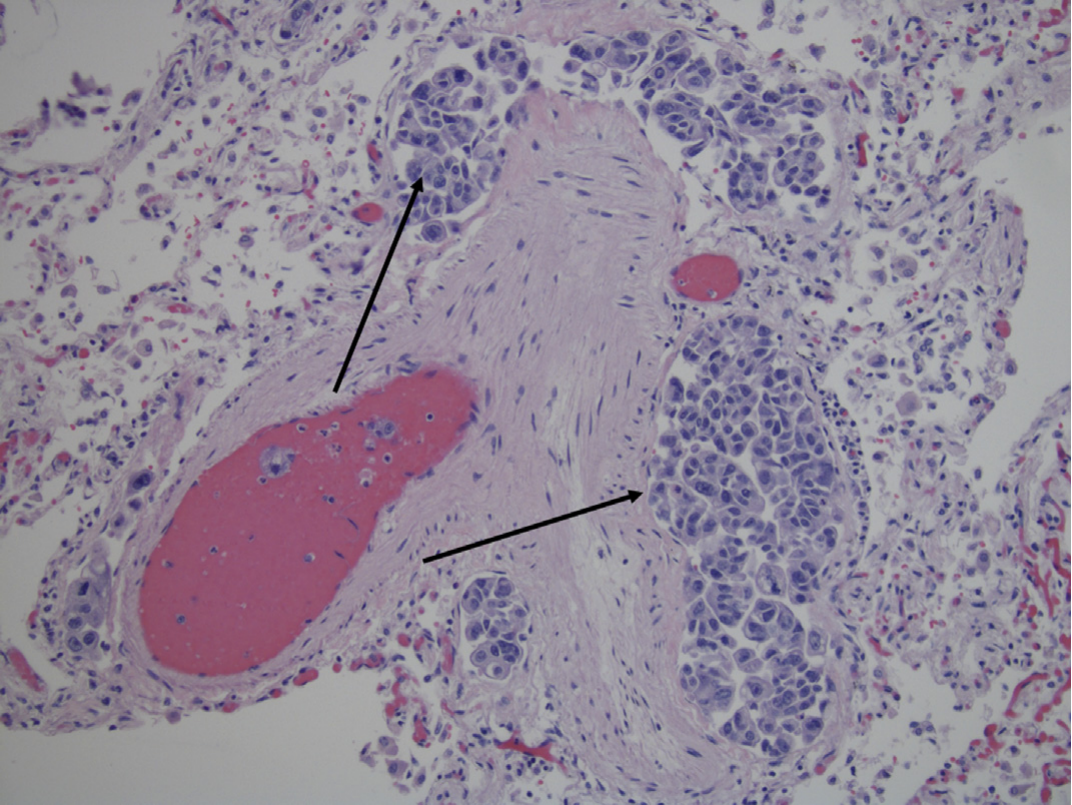
Pulmonary Vasculature Invasion by Intrahepatic Cholangiocarcinoma Section of lung, with tumor cells in veins and lymphatic vessels **(arrows)**.

Autopsy revealed metastatic intrahepatic cholangiocarcinoma with extensive tumor invasion of the cardiac and pulmonary vasculature and interstitium (Figure 1). These findings were consistent with pulmonary tumor thrombotic microangiopathy (PTTM), a universally fatal condition in which pulmonary arterial hypertension develops secondary to tumor microemboli.

## Discussion

Our case is only the third documented instance of PTTM occurring secondary to cholangiocarcinoma (1). The most commonly implicated malignancies in PTTM are gastric (59%), breast (10%), and lung (6%) (1,2).

PTTM develops as a result of nonocclusive tumor microemboli in the pulmonary vasculature, leading to endothelial damage, activation of the coagulation cascade, and release of growth factors and cytokines that result in obliterative intimal proliferation and ultimately pulmonary hypertension (1,2). Antemortem diagnosis is challenging and uncommon, but may be made by cytological analysis of the aspirate from a wedged pulmonary artery catheter (2). Mean survival time is <6 months from symptom onset (1). However, emerging evidence suggests pulmonary hypertension resulting from PTTM may be improved by the platelet-derived growth factor receptor tyrosine kinase inhibitor imatinib (2)*.*

## Funding Support and Author Disclosures

The authors have reported that they have no relationships relevant to the contents of this paper to disclose.

## Appendix

Supplemental Video 1 Echocardiogram Demonstrating Severe Right Ventricular Dilatation and Systolic Failure
